# Individual class evaluation and effective teaching characteristics in integrated curricula

**DOI:** 10.1186/s12909-017-1097-7

**Published:** 2017-12-12

**Authors:** Jung Eun Hwang, Na Jin Kim, Meiying Song, Yinji Cui, Eun Ju Kim, In Ae Park, Hye In Lee, Hye Jin Gong, Su Young Kim

**Affiliations:** 1 Department of Pathology, College of Medicine, The Catholic University of Korea, Seoul, Republic of Korea; 2 Master Center for Medical Education Support, College of Medicine, The Catholic University of Korea, 222 Banpo-daero, Seocho-gu, Seoul, 06591 Republic of Korea

**Keywords:** Academic achievement, Class evaluation, Effective teaching characteristics, Integrated curriculum, Undergraduate medical education

## Abstract

**Background:**

In an integrated curriculum, multiple instructors take part in a course in the form of team teaching. Accordingly, medical schools strive to manage each course run by numerous instructors. As part of the curriculum management, course evaluation is conducted, but a single, retrospective course evaluation does not comprehensively capture student perception of classes by different instructors. This study aimed to demonstrate the need for individual class evaluation, and further to identify teaching characteristics that instructors need to keep in mind when preparing classes.

**Methods:**

From 2014 to 2015, students at one medical school left comments on evaluation forms after each class. Courses were also assessed after each course. Their comments were categorized by connotation (positive or negative) and by subject. Within each subject category, test scores were compared between positively and negatively mentioned classes. The Mann-Whitney U test was performed to test group differences in scores. The same method was applied to the course evaluation data.

**Results:**

Test results for course evaluation showed group difference only in the *practice/participation* category. However, test results for individual class evaluation showed group differences in six categories: *difficulty*, *main points*, *attitude*, *media*/*contents*, *interest*, and *materials*. That is, the test scores of classes positively mentioned in six domains were significantly higher than those of negatively mentioned classes.

**Conclusions:**

It was proved that individual class evaluation is needed to manage multi-instructor courses in integrated curricula of medical schools. Based on the students’ extensive feedback, we identified teaching characteristics statistically related to academic achievement. School authorities can utilize these findings to encourage instructors to develop effective teaching characteristics in class preparation.

## Background

Team teaching is a widely used teaching method in integrated curricula. This method is one of the unique features of medical education, in which each course consists of a series of classes run by multiple instructors who majored in medical subjects [1, 2]. The advantage of this method is that each instructor conveys his or her specialized knowledge to medical students. However, students must adapt to the different styles of each instructor [3]. In addition, if deviations exist in instructors’ teaching styles, these differences can affect academic productivity [4]. Taking this into account, researchers have shown increased interest in integrated curriculum management to maintain consistency across courses [5, 6].

Given the team teaching practice in integrated curricula, a single course evaluation cannot capture students’ perceptions of classes taught by multiple faculty members. One reason individual class evaluation is required is that, because there are differences in instructors’ teaching methods and proficiency levels [7], the results of course evaluations cannot include students’ perceptions of all instructors or classes. Another reason is that it is practically impossible for students to remember and comment on multiple instructors’ classes. Thus, the course evaluation, which has been used so far, has limitations in properly assessing the integrated curriculum.

One of the intended goals of medical school lectures is to enhance academic achievement. It was reported that medical students expected to acquire facts through lectures [8]. Schools also expect students to have medical expertise. Therefore, in this study, we tried to find effective teaching characteristics in class by examining which areas of class evaluation were related to academic achievement. Previous research has reported that medical student satisfaction with a seminar was not correlated with academic achievement [7]. However, the study did not present an operational definition of satisfaction with the seminar. Readers could not discern the criteria used in the course evaluation. Another limitation of this approach was that, since the research setting was a discussion seminar, it was difficult to generalize the result to a lecture setting, which is common at Korean medical schools. More recently, another investigation examined the effects of team teaching on medical student performance [9]. Students who participated in team teaching sessions responded that this approach improved their comprehension and concentration and enhanced their interest in the topics. Because measurement of performance was based on students’ subjective responses, it could not be determined whether team teaching was related to objective academic performance.

The present study qualitatively analyzed students’ comments about each class. The reason for the qualitative analysis is to reduce errors when they reply to each item with the same answers [10], and to recognize strengths or weaknesses of instructors who influenced students’ learning processes. Several attempts were made to identify critical features of effective instructors. Ullian and colleagues [11] reported that students considered ten categories important for clinical instructors, including knowledge and clinical competence. Lin [12] also analyzed behaviors of problem-based learning tutors and reported that medical students preferred instructors with tutoring skills, medical and clinical knowledge, and positive personality. A qualitative study in Korea analyzed important characteristics of excellent lecturers at one medical school [13]. In the study, adequately summarizing learning content was regarded as the most important characteristic of a good lecturer. Some studies were carried out on excellent instructor’s features; however, to date, no studies have been conducted that investigated the association between students’ subjective comments on a class and the objective outcomes of the class.

The aim of this study was to establish the necessity of individual class evaluation and to confirm teaching characteristics related to academic achievement. For this, the authors qualitatively classified students’ comments on classes by connotation (positive or negative) and by subject, and analyzed if the test scores of positively mentioned classes were higher than those of negatively mentioned classes within each subject category. The results for class evaluation were compared to those for course evaluation. We hope that this research supports medical schools in managing the quality of classes in integrated curricula, and instructors to recognize the things to consider in class preparation.

## Methods

Basic medical education curriculum of Catholic University, College of Medicine in Seoul, Republic of Korea consists of a four-year program. Excluding clinical clerkship, 47 courses and 3329 classes are taught each year. The median number of classes and instructors per course is 49 (range, 15 to 135) and 17 (range 3–71), respectively. The median number of students per class is 95. Teaching methods in our curriculum included clinical clerkship, lecture, practice, case discussion, team-based learning (TBL), and student presentation, etc. Except for clinical clerkship, the most commonly used teaching method is lecture (71%). The teaching methods in each course showed similar distributions. Out of all courses from the first to the fourth year, we excluded fourteen courses that did not use computer-based test (CBT) as well as clinical clerkship, because it was not possible to calculate scores for each class in non-CBT classes. Accordingly, thirty-three courses were chosen for statistical analysis.

Two evaluation instruments were used to collect students’ evaluations of both classes and courses (Table 1). The items were developed by our school’s integrated curriculum committee of medical education specialists. The evaluation tools were composed of Likert scale ratings and open-ended questions. During 2014 and 2015, class assessments were completed online within 24 h after conclusion of the classes. Courses were evaluated within one week after completion. Class evaluations and course evaluations were conducted before students identified their scores so that knowing their scores did not affect class or course evaluations. It was assumed that because course evaluation was conducted at the end of courses, it would not be easy for students to recall all classes retrospectively. Thus, students were encouraged to comment on each instructor in class evaluations.

**Table 1:** Course and class evaluation tool

No.	Item	Not at all	A little	Moderately	Quite a bit	Extremely
A. Course evaluation						
1	Overall, I am satisfied with this course.	1	2	3	4	5
2	Course orientation was helpful: instructional plan including learning outcomes (objectives) and teaching methods	1	2	3	4	5
3	Evaluation criteria and evaluation methods were clearly conveyed in advance.	1	2	3	4	5
4	Materials (textbook, learning materials, etc.) helped to understand the contents of classes.	1	2	3	4	5
5	Classes were reasonably organized: all activities such as lectures and practices.	1	2	3	4	5
6	A holistic and integrated understanding of this course was possible.	1	2	3	4	5
7	The amount of contents covered in this course was appropriate.	1	2	3	4	5
8	The overall level of instruction in this course was appropriate.	1	2	3	4	5
9	Teaching methods (lectures, practices, presentations, and discussions, etc.) were effective for learning.	1	2	3	4	5
10	The exams adequately reflected the contents of classes.	1	2	3	4	5
11	What is the percentage of classes that guaranteed 10 min of rest after 50 min of classes?	0–20	20–40	40–60	60–80	80–100
12	I prepared for these classes in advance.	1	2	3	4	5
13	I participated in these classes faithfully.	1	2	3	4	5
14	I have fully reached the learning outcomes of this course/classes.	1	2	3	4	5
15^a^	A good thing about this course (open-ended):					
16^a^	What needs improvement (open-ended):					
B. Class evaluation						
1	The learning outcomes presented by the instructor were clear.	1	2	3	4	5
2	The amount of contents covered in this class was appropriate.	1	2	3	4	5
3	The level of instruction was appropriate to understand.	1	2	3	4	5
4	The teaching method helped me to achieve the learning outcomes.	1	2	3	4	5
5	The instructor’s class motivated me to learn about medicine.	1	2	3	4	5
6^a^	What do you think was good and what should be improved in this class? (less than 50 characters)					

^a^Responses to open-ended questions were used in this study

Students’ comments in each evaluation were manually reviewed and grouped by connotation and subject. The comments had positive, negative, or neutral connotations. Positive comments were encoded as 1, and negative comments were encoded as 0. We identified 19 themes in their comments (Table 2). Brief descriptions of the categories are as follows. *Evaluation* consists of comments on assignments and exams. *Schedule* includes class arrangement within a course and class-hour management, and *workload/pace* covers academic workload and pace of progress. *Difficulty* refers to whether an instructor easily explained contents. *Punctuality* reflects whether an instructor observed class hours, and *main points* indicates if learning objectives were clearly conveyed. *Speaking* refers to an instructor’s voice volume and speed of speaking. *Attitude* refers to an instructor’s attitude toward students, and *media/contents* involves effects of utilizing media and clinical cases. *Practice/participation* includes degree of student participation in simulations and discussions. *Faculty* consists of comments on coherence of classes by instructors, and *interest* covers how interesting a course or class was. *Communication* refers to degree of interaction between an instructor and students, *teaching methods* involves effects of class activities, and *materials* is about whether an instructor uploaded the materials in advance and requests to improve materials. *Physical environment* includes the physical characteristics of classroom settings. *General comments* include overall remarks such as “Thank you.” *Preparation* covers an instructor’s readiness for a course or class; finally, *quizzes* was about effectiveness and difficulty of quizzes.

**Table 2:** Frequency of students’ comments across categories

Category	Frequency (course)				Frequency (class)			
	Total (%)		Positive	Negative	Total (%)		Positive	Negative
Evaluation	70	(7.3)	0	70	39	(0.9)	0	39
Schedule	169	(17.7)	57	112	94	(2.1)	24	70
Workload/pace	74	(7.7)	4	70	387	(8.8)	37	350
Difficulty	49	(5.1)	14	35	710	(16.2)	443	267
Punctuality	7	(0.7)	1	6	250	(5.7)	12	238
Main points	71	(7.4)	47	24	593	(13.5)	440	153
Speaking	2	(0.2)	0	2	328	(7.5)	16	312
Attitude	38	(4)	36	2	101	(2.3)	84	17
Media/contents	125	(13.1)	97	28	573	(13.1)	518	55
Practice/participation	80	(8.4)	58	22	13	(0.3)	8	5
Faculty	9	(0.9)	5	4	4	(0.1)	0	4
Interest	62	(6.5)	62	0	585	(13.4)	572	13
Communication	4	(0.4)	4	0	58	(1.3)	53	5
Teaching methods	9	(0.9)	0	9	7	(0.2)	2	5
Materials	47	(4.9)	4	43	316	(7.2)	41	275
Physical environment	4	(0.4)	1	3	38	(0.9)	0	38
General comments	127	(13.3)	115	12	268	(6.1)	265	3
Preparation	2	(0.2)	0	2	3	(0.1)	0	3
Quizzes	6	(0.6)	1	5	15	(0.3)	11	4
Total	955	(100)	506	449	4382	(100)	2526	1856

During each course, student academic achievement was assessed with several exams. Most exams were online, structured as CBT containing multiple choice questions (MCQs). Properly structured MCQs clearly reflect predefined learning objectives and assess students’ abilities from knowledge to problem-solving [14], so test scores in this study means the degree of achievement of learning objectives. In addition, MCQs were used as a primary student evaluation tool in our basic medical education curriculum. Because of difficulty of standardizing test results, only MCQs were used as the evaluation tool for academic achievement. To produce score for class, we divided the MCQs used in a course’s exams by the corresponding classes, and re-calculated an average score for the class. Course score was an average score for exams taken during the course. The scores were presented as percent.

To determine significant associations between positive-negative comments and scores, we applied a few statistical tests to the data. First, we tested if students’ positive and negative comments were associated with their scores in classes and courses. Furthermore, after dividing the comments into categories, we verified that positive and negative comments within each category were related to the scores in classes and courses. Data distribution was assessed by the Kolmogorov-Smirnov test and the Shapiro-Wilk test. The score variable did not satisfy the normality. Thus, differences in scores between positively and negatively evaluated classes/courses were tested using the Mann-Whitney U test, with significance noted at *p* < 0.05. SPSS 19 software was used for analysis.

The study protocol (MC15EISI0121) was approved by the institutional review board of the Catholic University of Korea, College of Medicine. In accordance with the Declaration of Helsinki and its later amendments, all personal information was anonymized.

## Results

### The descriptive data

From 2014 to 2015, 79.3% of students completed class evaluations. Of the 43,109 class evaluations, 7832 comments (18.2%) were made. In terms of course evaluations, 70.8% of students responded. Of the 2847 course evaluations, 1714 comments (60.2%) were provided. The median scores of students who did not comment and who commented on classes were 80% and 65%. Mann-Whitney U test showed a significant difference in the scores of two groups (*P =* 0.000). For course evaluations, the median scores of students who did not comment and who commented were 77.4% and 76.8%. The test of Mann-Whitney U showed a significant difference in the scores (*P =* 0.047). The statistical difference in course evaluations might be due to the large sample size. Of the 7832 comments on classes, we excluded 528 items that were classified as neutral comments. Besides, 2922 items from non-CBT classes that did not produce scores were excluded. Finally, 4382 items were used for statistical analysis. The same criteria were applied to course evaluation data, so 955 of 1714 items were used for analysis. Although the rates of leaving comments were low, there was a reason to investigate them. We found that students’ survey fatigue from Likert scale rating increased with time, so all the results from Likert scale rating could not be used. Yet, making comments was less affected by survey fatigue than Likert scale rating.

**Fig. 1:**
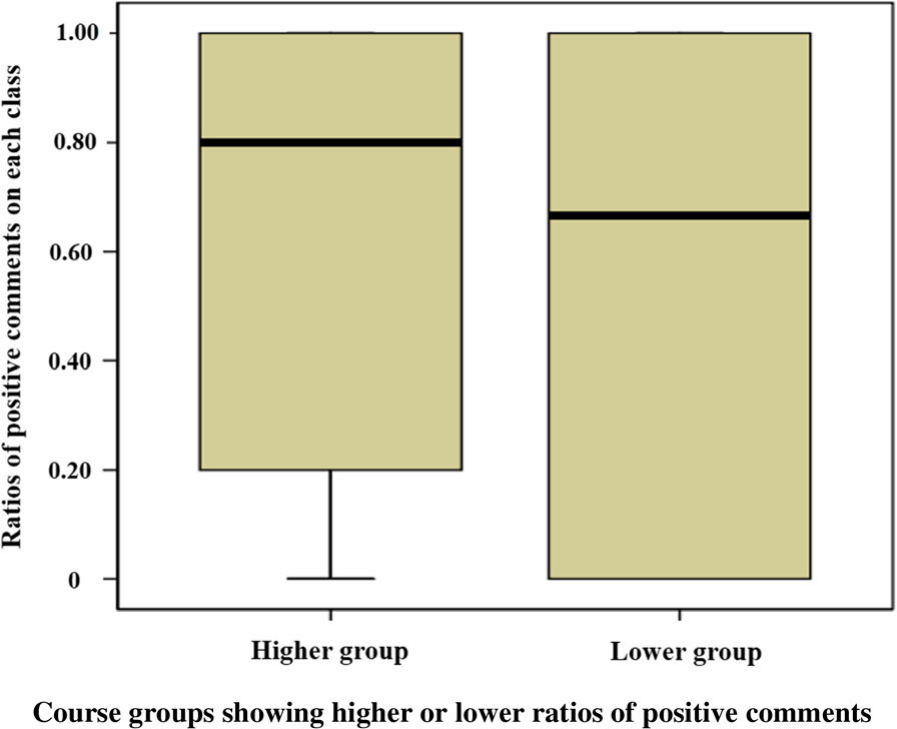
Distribution of ratios of positive comments on each class in higher and lower groups

Ratios of positive comments to total comments on each course were calculated, and we divided a higher group and a lower group by an average (0.52) of the ratios. In the same way, ratios of positive comments to total comments on each class were made. Figure 1 showed distribution of ratios of positive comments on each class in the higher and the lower group. Classes with higher ratios of positive comments were found in the lower group as well as in the higher group. However, the lower group had more classes with lower ratios of positive comments.

### The categorization of comments

As shown in Table 2, students made the most comments in course evaluations in order of *schedule*, *general comments*, *media*/*contents*, *practice*/*participation* categories. In class evaluations, on the other hand, the most comments were made in order of *Difficulty*, *Main points*, *Interest*, *Media/contents*.

The distribution of positive and negative comments by teaching methods was illustrated in Table 3. The Chi-square test result showed that students’ positive or negative comments were significantly related to types of teaching methods. Additionally, we conducted Kruskal-Wallis test to determine if comments on specific categories were indicative of test scores. The test results showed that there were significant differences in test scores across different category groups (*P* = 0.000).

**Table 3:** Chi square test result between students’ comments and teaching methods

Comment	Teaching methods				
	Lecture	Practice	Case discussion	Others^a^	χ^2^
Positive	3351 (59.1%)	25 (61.0%)	45 (63.4%)	31 (86.1%)	χ^2^ = 11.32 *P* = 0.010^*^
Negative	2316 (40.9%)	16 (39.0%)	26 (36.6%)	5 (13.9%)	
Total	5667 (100%)	41 (100%)	71 (100%)	36 (100%)	

^*^ *P* < 0.05

^a^Others: TBL, student presentation, etc.

### Identifying effective teaching characteristics in course and class

Table 4 provides results of the Mann-Whitney U test between positively and negatively mentioned courses/classes. Classes with positive descriptions exhibited higher class scores compared to classes with negative descriptions (*P* = 0.043). The same was true for course evaluation data. There was a significant difference in course scores between positively and negatively mentioned courses (*P* = 0.000).

**Table 4:** The results of the Mann-Whitney U test between positively and negatively mentioned groups

	Course			Class		
Scores	Positive^a^ (*n* = 506)	Negative^b^ (*n* = 449)	Mann-Whitney U test	Positive^c^ (*n* = 2526)	Negative^d^ (*n* = 1856)	Mann-Whitney U test
Median	78%	77%	*U* = 105,017 *P* = 0.043^*^	80%	72%	U = 1,790,363.5 *P* = 0.000^*^
Mean	76%	75%		78%	71%	
Min.	57%	57%		12%	6%	
Max.	91%	91%		100%	100%	

*P < 0.05

^a^positively commented courses

^b^negatively commented courses

^c^positively commented classes

^d^negatively commented classes

**Fig. 2:**
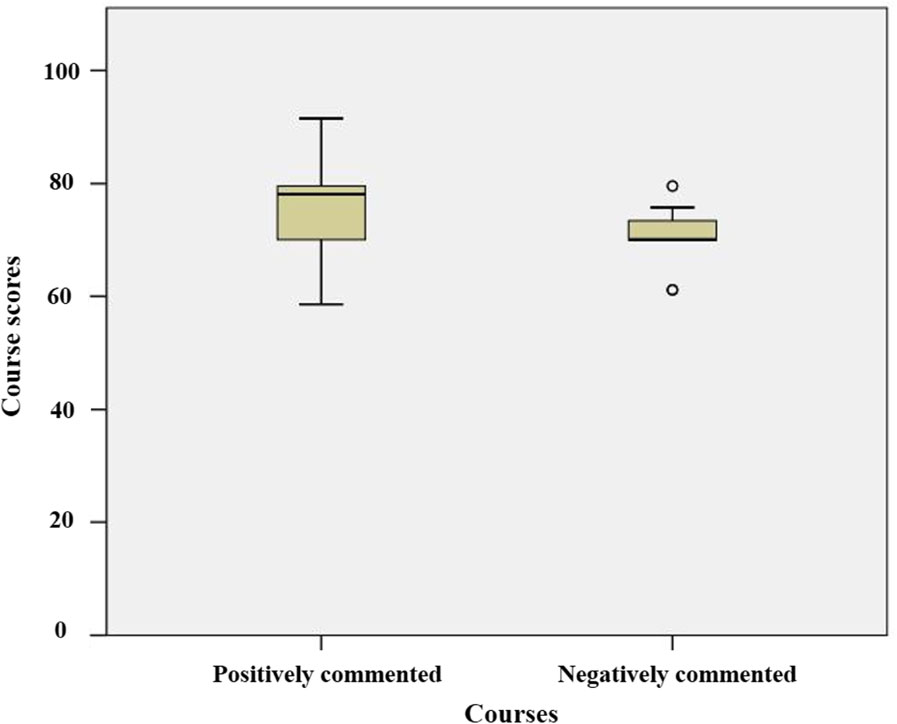
Score distribution of positively and negatively mentioned courses (*practice/participation*)

**Fig. 3:**
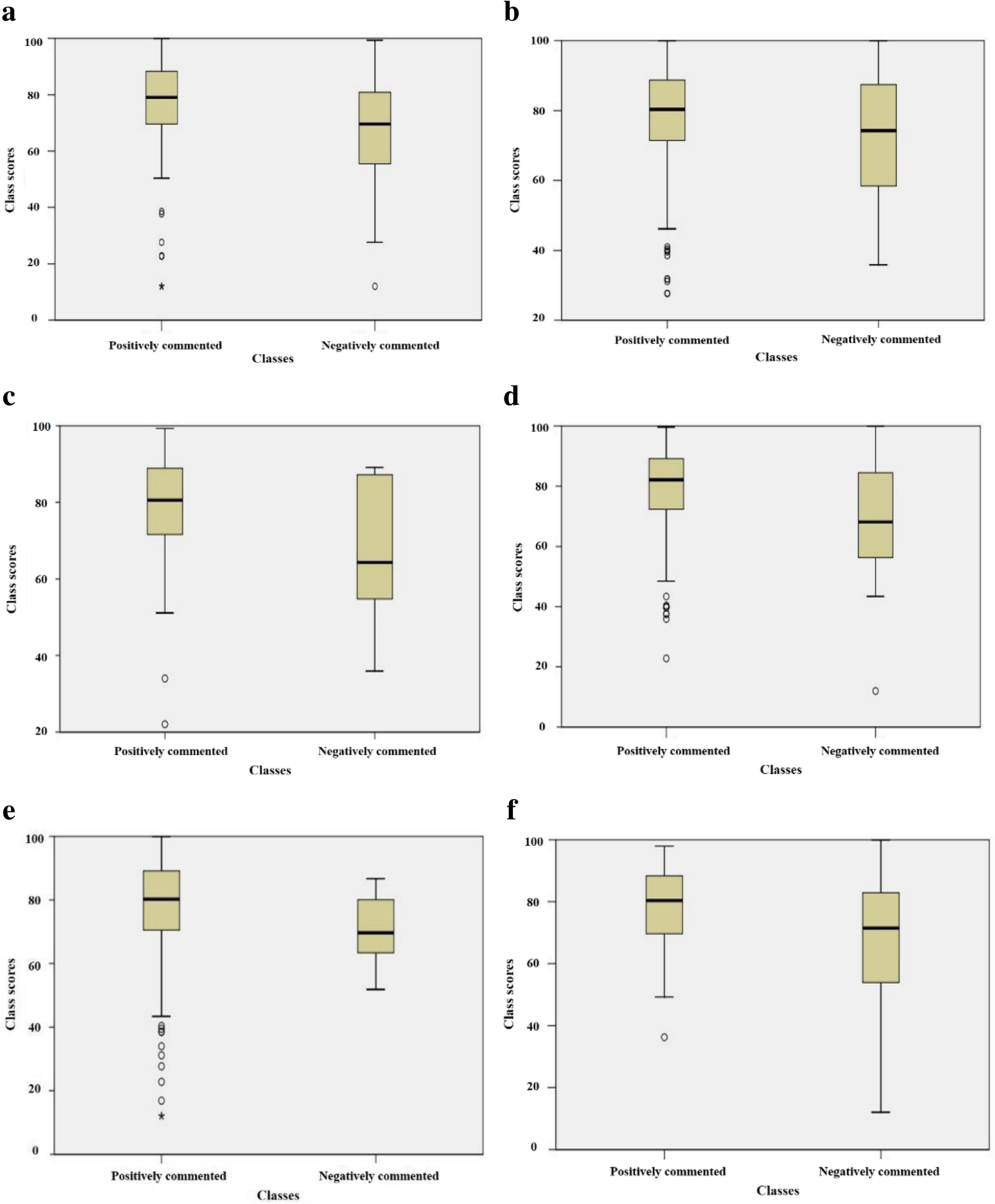
Score distribution of positively and negatively mentioned classes in six significant categories. **a** Difficulty. **b** Main points. **c** Attitude. **d** Media/contents. **e** Interest. **f** Materials

Test results for each subject category revealed that effective teaching characteristics in class differed from effective teaching characteristics in course. As shown in Table 5 and Fig. 2, only the *practice/participation* category in course evaluation indicated a significant difference in course scores (*P* = 0.005). In the other categories, course scores were not statistically different between positively and negatively described courses. In contrast, analysis of the class data showed that significant differences in class scores existed in six categories (Table 5, Fig. 3): *difficulty* (Fig. 3a; *P* = 0.000), *main points* (Fig. 3b; *P* = 0.001), *attitude* (Fig. 3c; *P* = 0.04), *media/contents* (Fig. 3d; *P* = 0.000), *interest* (Fig. 3e; *P* = 0.022), and *materials* (Fig. 3f; *P* = 0.01). Table 6 provides examples of students’ comments on the six significant subjects. The five categories of *schedule*, *workload/pace*, *speaking*, *communication*, and *teaching methods* showed slight differences in class scores but were not statistically significant.

**Table 5:** Comparison of scores in a positively and negatively mentioned courses/classes across categories

Category	Course			Class		
	Positive^a^	Negative^b^	Mann-Whitney U test	Positive^c^	Negative^d^	Mann-Whitney U test
Evaluation	-^e^	79%	N/A	–	71%	N/A
Schedule	79%	78%	*U* = 2893 *P* = 0.319	77%	73%	*U* = 812 *P* = 0.808
Workload/ pace	81%	76%	*U* = 65 *P* = 0.74	80%	71%	*U* = 5435.5 *P* = 0.107
Difficulty	77%	79%	*U* = 206.5 *P* = 0.393	79%	70%	*U* = 43,151.5 *P* = 0.000^*^
Punctuality	80%	77%	*U* = 0 *P* = 0.286	75%	79%	*U* = 1290.5 *P* = 0.573
Main points	78%	78%	*U* = 517.5 *P* = 0.571	80%	74%	*U* = 27,722 *P* = 0.001^*^
Speaking	–	76%	N/A	78%	73%	*U* = 1976 *P* = 0.16
Attitude	73%	80%	*U* = 14.5 *P* = 0.182	81%	64%	*U* = 488 *P* = 0.04^*^
Media/contents	79%	78%	*U* = 1180.5 *P* = 0.292	82%	68%	*U* = 8949 *P* = 0.000^*^
Practice/participation	78%	70%	*U* = 383.5 *P* = 0.005^*^	64%	85%	*U* = 10 *P* = 0.143
Faculty	73%	78%	*U* = 7 *P* = 0.556	–	96%	N/A
Interest	77%	–	N/A	80%	70%	*U* = 2340.5 *P* = 0.022^*^
Communication	76%	–	N/A	72%	66%	*U* = 86 *P* = 0.196
Teaching methods	–	70%	N/A	71%	62%	*U* = 3 *P* = 0.434
Materials	79%	76%	*U* = 65.5 *P* = 0.449	80%	71%	*U* = 4226 *P* = 0.01^*^
Physical environment	70%	70%	*U* = 1.5 *P* = 1	–	71%	N/A
General comments	78%	75%	*U* = 514 *P* = 0.146	82%	87%	*U* = 275 *P* = 0.359
Preparation	–	68%	N/A	–	67%	N/A
Quizzes	78%	78%	*U* = 2 *P* = 1	77%	86%	*U* = 13 *P* = 0.238

*P < 0.05

^a^positively commented courses

^b^negatively commented courses

^c^positively commented classes

^d^negatively commented classes

^e^The number of comments was 0

**Table 6:** Examples of students’ comments on the six significant subjects

Subject	Comments	
	Positive	Negative
Difficulty	♦ When the instructor taught, s/he spoke slowly and explained things well. ♦ The instructor’s explanation was clear and easy to understand.	♦ I wished the instructor explained more clearly and slowly. ♦ In my opinion, the instructor’s explanation was insufficient.
Main points	♦ Because the instructor emphasized and repeated the important contents, I was able to learn new contents. ♦ I appreciated that the instructor helped to distinguish what students should know and the details.	♦ I wanted the instructor to put more emphasis on main points. ♦ If the instructor had presented the learning objectives clearly, it would have been a better lecture.
Attitude	♦ The instructor was enthusiastic. ♦ The instructor’s consideration for students was apparent.	♦ Students learned this content for the first time, but the instructor did not seem to consider our level. ♦ The instructor needs to recognize that his/her statements can negatively impact students.
Media/contents	♦ It was good that the instructor explained numerous clinical cases. ♦ Looking at real photos and videos in class helped me remember them longer.	♦ Non-scientific contents did not seem to be useful for medical students. ♦ I wanted the instructor to explain in more detail how students could diagnose cases.
Interest	♦ The contents of slides were impressive and interesting. ♦ The instructor made the class fun.	♦ The class was a little monotonous. ♦ It was a little boring.
Materials	♦ The slides were simple and clear, and the structure was easy to understand. ♦ Class materials were uploaded before class started.	♦ It was difficult to understand the contents because I did not receive class materials. ♦ The indications on the slides were incorrect, so it was hard to follow the instructor.

## Discussion

The present study was set out to prove the necessity of class evaluation in integrated curricula and to identify effective teaching characteristics in class. The results of this study supported the following conclusions.

Students’ evaluations of multiple-instructor courses in a single, retrospective manner might not adequately represent their true experience in classes. The importance of class evaluation was confirmed in two aspects. First, effective teaching characteristics in class differed from those in course. For course evaluation, it was only one category that was associated with students’ scores, but six categories in class evaluations were significantly related to class scores. This result is in line with that of a preliminary study that compared prospective class assessment and retrospective course assessment [15]. In the study, the researchers concluded that evaluation immediately after classes was higher than retrospective course evaluation in quality, since medical students could not remember and rate every class in course evaluation. Similarly, the course evaluation data in this study showed that many students mentioned the most memorable point that was good, rather than providing specific comments on classes that consisted of the course. Second, the fact that classes with higher ratios of positive comments were found within the lower ratio course group suggests that a course may be negatively evaluated by a handful of classes with lower ratios of positive comments (Fig. 1). This interpretation cannot be derived from course evaluation alone.

According to previous studies [1, 16], course evaluation has typically been conducted at the end of courses. Findings of this study, however, imply that continuous class evaluation is a practical tool for integrated curriculum management. Recently, researchers have begun to describe their experiences with an integrated curriculum at medical schools [5, 6]. One medical school in the research has set one of its goals as effective curriculum management in the process of restructuring into integrated curriculum [5]. As part of this process, teaching activities and productivity were monitored through student comments, performance in exams, and class audits to sustain uniformity and harmony between courses and within a course. The medical school in this study also concluded that there was a limit to utilizing course evaluations to manage the quality of classes in an integrated curriculum. Through this research, we demonstrated quantitative and qualitative differences between class and course evaluations.

Second, key elements that instructors should keep in mind were identified to enhance learning outcomes. Classes that received positive comments from students in the categories of *difficulty*, *main points*, *attitude*, *media/contents*, *interest*, and *materials* produced higher academic achievement than classes that received negative comments.

The effects of *difficulty* and *main points* on academic achievement represent needs of medical students and show that these two factors are related to medical student academic achievement. The results of this study support previous research that investigated requisites for good teaching [17]. The study found that the most satisfactory type of lecture for medical students was that in which they could easily learn important contents. Students and professors answered that if they had the opportunity to attend other lectures, they would see whether the lecture would make sure students knew main points. Results of the current study also reveal that medical students want instructors to easily and clearly explain knowledge, and that if instructors fail to control the difficulty of classes and deliver main points, student academic achievement can be affected.

In high-scoring classes, students’ positive comments on *media/contents* showed that contents were well remembered when students were exposed to related pictures, videos, and various clinical examples. These results are consistent with recent research results, suggesting use of multimedia and case presentations to increase the effects of teaching in medical schools [18, 19]. Further, the result can be explained by the multi-media theory. In other words, activation of information processing by utilizing multiple channels of visual/pictorial and auditory/verbal learning contents leads to meaningful learning [20].

The emotional domains such as attitude of instructors and degree of interest were found to be related to academic achievement. It has been reported that instructors’ respect for students and linguistic or nonverbal enthusiasm influenced pharmacological students’ potential vitality, inner motivation, and final course grades [21]. With respect to interest, comments from the high-scoring classes in this study included “the professor was witty,” and “the contents of the class were really interesting.” Whether the comments came from humor of instructors or from academic interest, the students in the classes seemed to focus indirectly on interesting objects and to acquire knowledge through exploratory activities with the objects [22].

Finally, students often left negative comments on class materials in the classes that received low scores. Comments included, “I wanted the professor to upload materials before the class starts,” or “I didn’t know what the professor was explaining because there were not indicators at all on the slides.” Because lectures are still one of the major instructional forms in medical schools [2, 8, 13, 23], presentation slides as a supplementary material needs to be properly designed according to students’ needs [23].

The results presented here should be interpreted in a setting of online MCQ evaluation. The researchers excluded data of classes that used something other than an online CBT system including MCQs. That is, it is difficult to say that effective teaching characteristics in class found in this study are valid in other classes that used essays and project evaluations. In addition, students’ neutral comments such as suggestions for the online system and for future directions were not included in the analysis. Thus, the results of this study do not reflect the opinions of all medical students in our school.

Notwithstanding these limitations, the present study demonstrated that effective teaching characteristics in class and those in course were different from each other, suggesting the need for individual class evaluation for quality control in an integrated curriculum. In addition, we confirmed six characteristics of classes related to medical student academic achievement.

## Conclusions

In integrated curricula, course evaluation may not comprehensively reflect student perception of the courses. From this research, it was found that individual class evaluation is required to manage courses run by multiple instructors. School authorities can use these findings to ensure that instructors have effective teaching characteristics for students to develop medical expertise through classes.

## Abbreviations

CBT: Computer-based test
MCQ: Multiple choice question
TBL: Team-based learning
